# Spatial Distribution of *Aedes aegypti* Oviposition Temporal Patterns and Their Relationship with Environment and Dengue Incidence

**DOI:** 10.3390/insects12100919

**Published:** 2021-10-09

**Authors:** Verónica Andreo, Ximena Porcasi, Claudio Guzman, Laura Lopez, Carlos M. Scavuzzo

**Affiliations:** 1 Instituto de Altos Estudios Espaciales “Mario Gulich”, Falda del Cañete, Córdoba 5187, Argentina; ximena.porcasi@conae.gov.ar (X.P.); scavuzzo@conae.gov.ar (C.M.S.); 2 Consejo Nacional de Investigaciones Científicas y Técnicas (CONICET), Ciudad Autónoma de Buenos Aires C1425FQB, Argentina; 3 Programa de Zoonosis, Área de Epidemiología, Ministerio de Salud, Córdoba 5000, Argentina; Cguzman77@hotmail.com (C.G.); laulop@hotmail.com (L.L.)

**Keywords:** mosquitoes, clustering, time series, Earth Observation, machine learning

## Abstract

**Simple Summary:**

*Aedes aegypti*, the mosquito species that transmits dengue virus among others, is fully adapted to thrive in urban areas. Their activity, however, varies in time and space and this might imply different transmission risk. We hypothesize that the temporal differences in mosquito activity are determined by local environmental conditions. Hence, we explore the existence of groups of temporal patterns in weekly time series of ovitraps records and we associate those patterns to environmental variables derived from remote sensing data and also to dengue incidence. We found three groups of temporal patterns that showed association with land cover diversity, heterogeneity and variability in vegetation and humidity indices estimated over 50-m radius buffer areas surrounding ovitraps. Dengue incidence on a neighborhood basis showed a weak but positive association with the percentage of pixels belonging to one of the patterns detected. The understanding of the spatial distribution of temporal patterns and their environmental determinants might then become relevant to guide the allocation of prevention and monitoring interventions.

**Abstract:**

*Aedes aegypti*, the mosquito species transmitting dengue, zika, chikungunya and yellow fever viruses, is fully adapted to thrive in urban areas. The temporal activity of this mosquito, however, varies within urban areas which might imply different transmission risk. In this work, we hypothesize that temporal differences in mosquito activity patterns are determined by local environmental conditions. Hence, we explore the existence of groups of temporal patterns in weekly time series of *Ae. aegypti* ovitraps records (2017–2019) by means of time series clustering. Next, with the aim of predicting risk in places with no mosquito field data, we use machine learning classification tools to assess the association of temporal patterns with environmental variables derived from satellite imagery and predict temporal patterns over the city area to finally test the relationship with dengue incidence. We found three groups of temporal patterns that showed association with land cover diversity, variability in vegetation and humidity and, heterogeneity measured by texture indices estimated over buffer areas surrounding ovitraps. Dengue incidence on a neighborhood basis showed a weak but positive association with the percentage of pixels belonging to only one of the temporal patterns detected. The understanding of the spatial distribution of temporal patterns and their environmental determinants might then become highly relevant to guide the allocation of prevention and potential interventions. Further investigation is still needed though to incorporate other determinants not considered here.

## 1. Introduction

*Aedes aegypti* is the main vector of dengue, zika, chikungunya and yellow fever viruses worldwide. In recent decades, aided by global warming, urbanization, trade and human migration, *Ae. aegypti* has invaded many temperate areas of the world [1,2], reaching latitudes as south as 40° S [3]. This mosquito species is highly anthropophilic and it is fully adapted to thrive in urban areas, where it can fulfill all its ecological needs. Its success is mainly due to its behavior and survival ability, which includes egg laying in natural and man-made water containers associated with domestic and peridomestic settings and eggs’ resistance to desiccation [4]. These characteristics, plus a wide distribution and high densities of breeding sites, constitute the key factors that mainly determine the circulation and transmission of dengue and related viruses in urban areas [5].

Dengue fever causes the greatest human disease burden, with an estimated 10,000 deaths and 100,000 million symptomatic infections per year in over 125 countries (53% of the global population at risk) [6]. The incidence of dengue has grown dramatically in recent decades, with a concomitant increasing frequency of outbreaks, especially in South America during the past 10–12 years. Two of the most important dengue outbreaks in Argentina occurred in 2009 and 2016, affecting more than 25,000 and 40,000 people, respectively, and reaching temperate cities such as Córdoba and Buenos Aires [7,8,9,10]. During 2020, in the midst of COVID-19 pandemic, Argentina experienced the largest dengue outbreak to date, with more than 58,000 confirmed cases, and near 3000 in the city of Córdoba [11].

Given the lack of a well established and accepted vaccine, dengue control and prevention is traditionally based on vector control and entomological surveillance to estimate the potential risk for virus transmission and disease [12]. Prevention programs are therefore typically focused on removal of *Ae. aegypti* breeding sites in order to eliminate vector larval stages, treatment of larval habitats and insecticidal spraying to reduce adult density [13]. The latter being usually applied as a reactive measure once cases appear.

In this context, understanding the determinants of mosquito spatial distribution and temporal variations in abundance is key [14,15]. Indeed, many studies have focused on mapping vector hotspots and disease clusters, as well as understanding the causes of spatial heterogeneity at different scales as key tools for decision making regarding prevention programs and control actions [16,17,18,19,20]. Others have addressed the prediction of temporal changes in mosquito abundances, either through mechanistic [21] or empirical models [22,23,24] with the aim of forecasting risk. In most cases, Earth Observation (EO) data of different spatial and temporal resolution were used as the main source of environmental information to relate to mosquito data and yield predictions [14]. In a different path but with the same aim, various clustering techniques have been used, i.e., spatiotemporal clustering was applied to identify clusters of human cases during outbreaks [8,9], co-clustering techniques were used to identify favorable space-time conditions triggering outbreaks [25], spatial clustering was used in the identification of different cover types or groups of neighborhoods [26].

Time series clustering is a special type of clustering that handles dynamic data and has recently received more attention [27]. Examples in the literature commonly include the use of Dynamic Time Warping (DTW) distance combined with a clustering method to assess patterns in economic time series [28], crop classification [29], hydrodynamic behavior of ground water level [30] and phenological regions delineation [31]. Remarkably, only once has clustering been applied to time series of vector data in an attempt to understand if there might be different temporal patterns that could explain the spatial differences usually observed in timing of cases occurrence and transmission risk [32].

In this contribution, we explore a novel approach that combines time series clustering, EO data, machine learning and dengue incidence, to assess the existence and spatial distribution of *Ae. aegypti* oviposition temporal patterns and their relationship with environment and dengue incidence in the city of Córdoba. Under the hypothesis that local environmental features might determine differences in female mosquitoes oviposition patterns and that different temporal patterns might be related to different risk levels of dengue transmission, we use time series clustering to group time series of eggs’ counts. Furthermore, since our ultimate goal is to enhance operational tools and predict risk in places where we do not have data, we then assess the relationship between temporal patterns and environmental variables extracted from the analysis of high resolution (10 m) EO data in the surroundings of each ovitrap. This allows us to predict types of temporal patterns over space, which in turn facilitates the characterization of neighborhoods according to oviposition activity pattern and evaluate the relationship with dengue occurrence in the last outbreak.

## 2. Materials and Methods

### 2.1. Study Area

Córdoba is the second largest city in Argentina with a population of 1,330,023 inhabitants in 2010 [33]. It has a surface of 576 km${}^{2}$ and it is located at 31°24${}^{\prime }$ S, 64°11${}^{\prime }$ W, 450 m.a.s.l. The urban area is surrounded by agricultural fields and small forest patches.

Córdoba city has a temperate climate, with mean annual precipitation of 800 mm. The winter is markedly dry and most precipitation occurs in the summer months. The rainy season spans between October and March, with the highest precipitations from December to February. The mean annual temperature is 21 °C (range 12–38 °C). Winters are temperate, with several frost days in June and July.

The Suquía River, its tributary La Cañada and numerous additional water channels run through the city. Human activities have resulted in a landscape characterized by a highly developed urban core represented by buildings and green areas in the form of urban parks. Suburban areas are characterized by residential neighborhoods, primarily single-family houses with yards, interspersed with parks and other green spaces.

### 2.2. Mosquito Data

**Figure 1:**
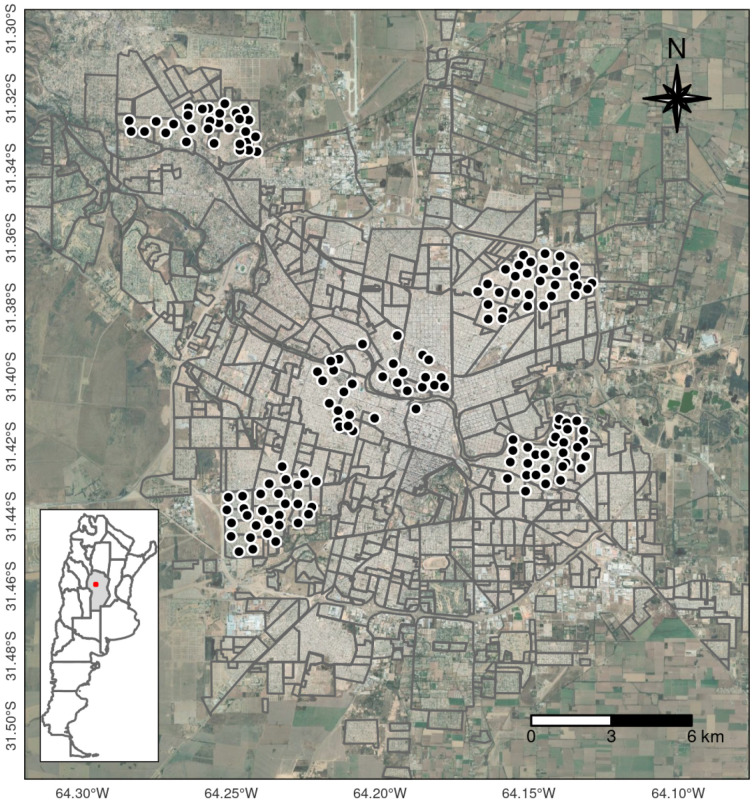
Distribution of ovitraps in Córdoba city (Argentina).

Entomological data consisted of 300 ovitraps distributed in 150 houses over 5 different areas of the city (Figure 1). Houses with ovitraps were at least 150 m apart, with an average distance of 350 m.

Two ovitraps were placed in the front yard of each house, usually in shaded places and below or close to bushes or pots with plants. The possibility of trap installation depended on householders’ written consent. Each ovitrap consisted of a black 1000 mL plastic container filled with 250 mL of water and a wooden tongue depressor (15 × 2 cm) held vertically to serve as substrate for mosquito oviposition [34]. Eggs laid in each wooden paddle were counted under magnifying glass. All ovitraps were replaced every week, from October 2017 to December 2019. Since the two ovitraps per house are not discriminated in the field, i.e., they are not labeled distinctively, we use the average count of eggs per house per week as the input for further analysis. We performed temporal linear interpolations when data were missing for different reasons. We removed houses (i.e., time series) with more than 10 consecutive missing values to avoid artifacts in temporal interpolations arising from not enough valid data points.

### 2.3. Remote Sensing Data

We used Sentinel 2 data for 3 different periods covering the core mosquito seasons in the area during our study period: November 2017 to March 2018, November 2018 to March 2019, and November 2019 to March 2020. A detail of the scenes processed can be found in Table S1 of the Supplementary Materials. Sentinel 2 imagery consists of 12 bands (3 visible and near infrared at 10 m, 3 red-edge at 20 m, 1 near infrared at 20 m, 2 short wave infrared at 60 m, among others). It has a revisit time of around 5 days, given that there are 2 satellites, Sentinel 2A and Sentinel 2B. We selected level 1 scenes with no clouds, one per month, in similar dates, for the 3 periods described above. All bands were atmospherically corrected by means of the Atmospheric and Radiometric Correction of Satellite Imagery (ARCSI) software and imported into GRASS GIS 7.8 [35] where all further remote sensing processing was done.

Common vegetation and water indices such as the Normalized Difference Vegetation Index (NDVI) and the Normalized Difference Water Index (NDWI) were derived for each image. Then, they were temporally averaged to obtain a single summary index image per season. We also estimated a synthetic panchromatic band for each season by temporally aggregating red, green and blue bands using the median (RGB composites for each season are shown in Figure S1 in the Supplementary Materials), and then, averaging them.

Following [20], a k-means unsupervised classification with 15 different classes was chosen as to identify different spectral covers (see Figure S2 in the Supplementary Materials). Buffers of ≈50- and 100-m radius from each ovitrap were overlaid upon bands and different statistics and texture measures were obtained, i.e., number of classes, the most common class, class diversity, mean and standard deviation (sd) NDVI, mean and sd NDWI, contrast, entropy, interspersion, etc. Buffer sizes were established according to the commonly used 100 m flight range of host-seeking female mosquitoes. All variables derived from remote sensing data are defined and explained in Table S2 in Supplementary Materials.

### 2.4. Dengue Data

Locations of imported and autochthonous dengue cases for the 2019–2020 season were provided by the Health authorities of Córdoba province. In total, there were 2755 dengue cases in Córdoba city in the 2019–2020 season, 28 were imported and 2727 autochthonous out of which we recovered 2491 coordinates (see Section 3).

### 2.5. Data Analyses

#### 2.5.1. Time Series Clustering

We tested different partitional time series clustering algorithms and different distance measures with number of clusters (k) ranging from 3 to 10 (Table 1) [27]. We ran 10 repetitions of maximum 100 iterations for each combination of k, distance, and centroid extraction method. For the case of Dynamic Time Warping (DTW) clustering, there were other parameters considered such as vector norm (i.e., Manhattan and Euclidean distances, L1 and L2, respectively) and window size (from 1 to 5, in steps of 1). Since the shape-based distance (SBD) requires data normalization, we applied this pre-processing step in all cases for the sake of consistency, and we tested configurations both with and without centering.

Since clustering is an unsupervised method, we used internal cluster validity indices (CVI) to evaluate the results. These indices only consider the partitioned data and try to quantify cluster purity. Each index defines its range of values and whether they are to be minimized or maximized to assign a vote to a certain configuration within a set [36]. Several CVIs were estimated for each combination of settings and majority vote was used to decide on a final result. The CVIs used were: Silhouette (Sil), Dunn (D), COP index (COP), Davies–Bouldin (DB) and Modified Davies–Bouldin (DB*) [36]. We compared all 6 combinations from Table 1 for 3 different periods: the full study period (117 weeks, October 2017–December 2019), season 2017–2018 (October 2017–September 2018) and, season 2018–2019 (October 2018–September 2019). We then performed majority vote among the 6 configurations per subset to select one best configuration for further comparisons. For time series clustering and evaluation of results, we used the *dtwclust* package [37] in the R software [38]. The results of the best configurations were then compared using different similarity measures from the *clue* package [39].

#### 2.5.2. Association with Environmental Features

To understand if local environmental variables derived from remote sensing in 50- and 100-m radius areas could explain the clustering of ovitraps time series obtained, we ran random forest (RF) classifications for each period. The dependent variable or outcome was the cluster number. These analyses were run with the package *caret* [40] in the R software [38]. In all cases, we split the data into training and test sets (70 and 30%, respectively). Given that clusters were unbalanced, we used an up-sample approach before RF training to increase n in smaller groups. Furthermore, we centered and scaled variables in the training sample to avoid that differences in values might affect variable importance. We used repeated cross-validation for hyper-parameter tuning in the training phase, with 5 folds and 10 repetitions. We performed variable selection by means of anova scores filter. This method uses anova *p*-values as weights to decide which variables or features are to be used in the classification step and we estimated variable importance to infer relationships with clusters. Finally, we evaluated the classification’s results with the 30% test dataset that was left aside at the beginning. Overall accuracy was used as performance measure both for training and validation.

Since the ovitrap sampling could not continue given the control activities that had to be performed because of the dengue outbreak starting in January 2020 all over the province, and the COVID-19 pandemic afterwards, we do not have complete ovitrap records for 2019–2020 as to carry out the analysis. Therefore, we used 2018–2019 RF model to predict the time series pattern over centered and scaled remotely sensed data for 2019–2020.

### 2.6. Oviposition Temporal Patterns and Dengue

In the attempt of uncovering potential associations among the occurrence of different oviposition temporal patterns and dengue cases, we aggregated data by neighborhood. To estimate dengue incidence over 10,000 people per neighborhood, we first estimated population count by adding up the 100-m resolution data UN adjusted produced by WorldPop (https://www.worldpop.org/, accessed on 30 June 2021) for 2020 in each neighborhood of the city of Córdoba. We then obtained the percentage of pixels belonging to different temporal patterns in each neighborhood. Afterwards, we compared incidences and percentage of each oviposition temporal pattern along all neighborhoods with at least 1 dengue case by means of Spearman rank correlation analysis.

All GRASS and R scripts used to perform data processing, analysis and visualization are available at: https://github.com/veroandreo/mosquito-ts-clust.

## 3. Results

**Figure 2:**
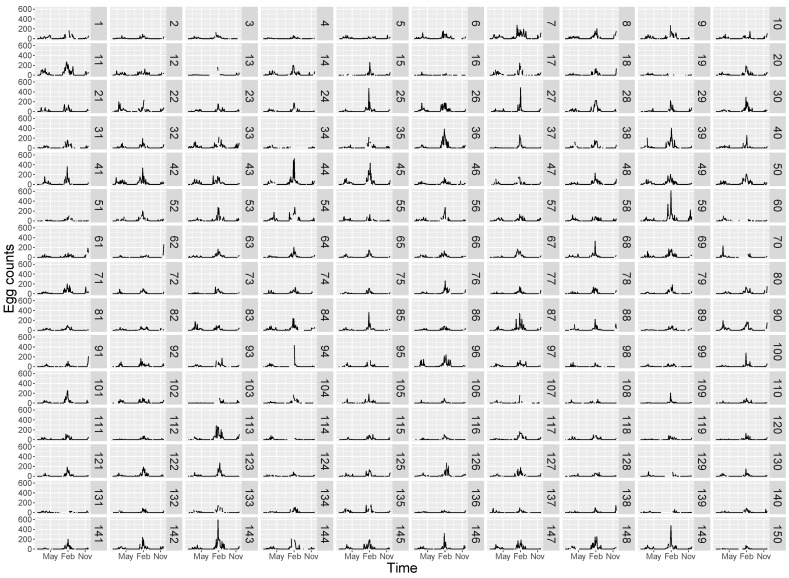
Time series plots of average egg counts as measured by 300 ovitraps distributed in 150 houses in Córdoba (Argentina). Period: September 2017–December 2019. Numbers 1 to 150 represent the houses where ovitraps were placed. Houses number 13, 19, 34, 60, 70, 131 and 139 were excluded of further analyses because they presented more than 10 consecutive time steps without records.

Average egg counts varied markedly both in time and space (Figure 2). There was, however, a seasonal pattern in oviposition with 2018–2019 season reaching generally much higher egg counts. The average maximum egg count was 70 in 2017–2018 season, while it reached 185 in the following season. Furthermore, the maximum egg count for a single household was 236 in 2017–2018 (house 70) and 615 in 2018–2019 (house 59). The median date of maximum egg count was only one week earlier in 2018–2019 compared to the previous season, 21 January vs. 29 January, respectively.

The earliest onset in oviposition was observed in week 42 (mid October) in 2017, 4 weeks earlier in 2018 (week 38, mid September) and in week 39 in 2019. On the other hand, oviposition stopped as late as week 22 in 2018 (by the end of May) and by mid-late June in 2019 (week 25). These onset and offset dates resulted in a reproductive season of more than 7 months in the period 2017–2018, and ≈9 months in 2018–2019.

**Figure 3:**
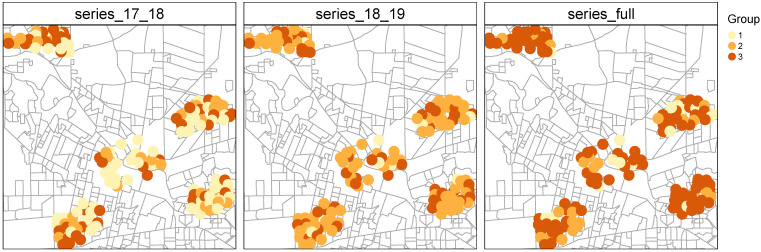
Spatial distribution of time series clustering results for the different periods studied. Left panel: season 2017–2018, central panel: season 2018–2019 and, right panel: full period 2017–2019.

From all the clustering configurations tested, we obtained the best distance-centroid combination for each period considered (Table 2). The selection of the best combination of parameters within each distance-centroid combination and period was made according to CVIs and majority vote. We then performed a second round of votes to obtain the best clustering configuration per period. All the best configurations split the 143 ovitraps time series in 3 groups (i.e., the minimum k tested). Two of the selected configurations are based on DTW clustering and the third one corresponds to K-shape clustering (SBD distance + shape extraction centroid). Both cluster sizes and cluster spatial distribution differed according to periods (Figure 3). Generally, however, the algorithms identified one large group and the other two much less so (Table 2).

The median end date of oviposition was pretty constant for all groups in the different clustering algorithms both in the period 2017–2018 and 2018–2019 (Table 3). Median starting date, however, was a bit more variable, determining longer or shorter oviposition seasons in each case. Remarkably, the median start date in 2018–2019 season was around one month earlier than in the previous season (Table 3). Hence, the median duration of oviposition season was ≈20–30 days longer in 2018–2019. The mean maximum number of eggs is 2 to 3 times higher in 2018–2019 than in the previous season, while the median date of the peak is approximately the same.

**Figure 4:**
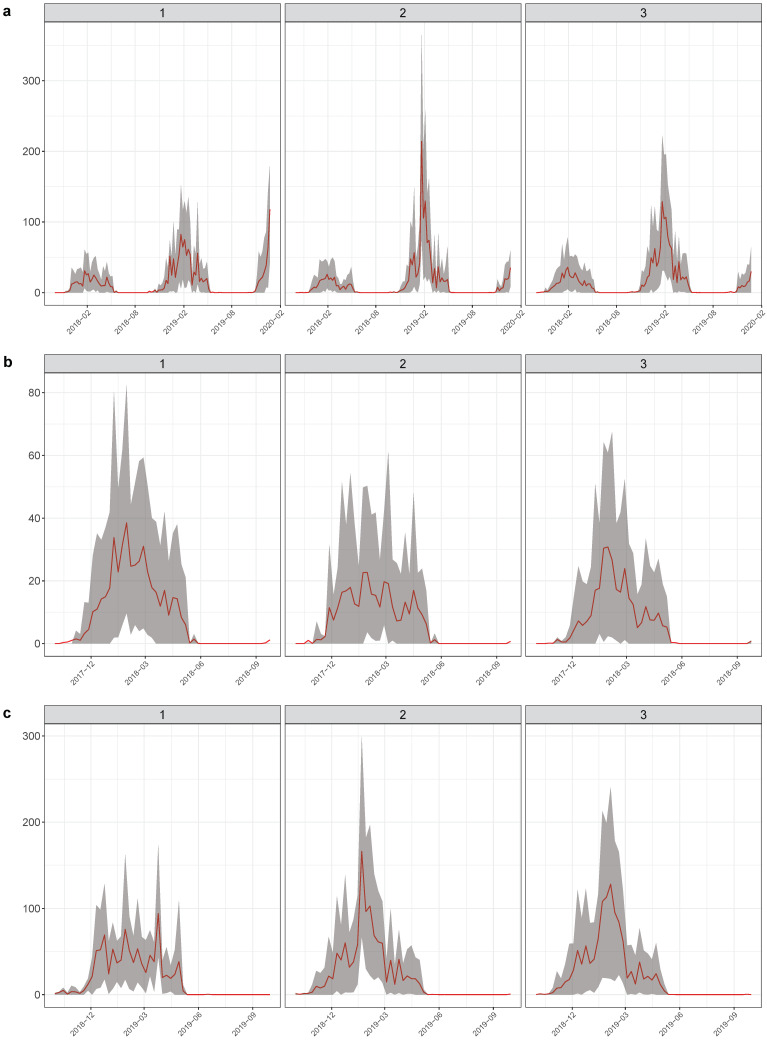
Mean egg count (red line) of the clusters obtained for (**a**) the full series, (**b**) 2017–2018 and (**c**) 2018–2019. Clustering groups are in columns. The gray area represents the 10th and 90th percentiles.

The difference among seasons was also evident in the plots showing mean egg counts and 10 and 90 percentiles for each cluster in the three time periods considered. Egg counts were much higher in 2018–2019 than the year before (Figure 4). Statistically, however, only in 2017–2018 were there significant differences among clusters in terms of egg counts.

Regarding the shape of the curves, both in 2017–2018 and 2018–2019, there seems to be a temporal pattern with lower abundances and no clear peak (group 2 in 2017–2018 and group 1 in 2018–2019, Figure 4). In 2017–2018, among the groups with the highest egg counts, group 1 appears to maintain higher abundances over a longer period than group 3 (Figure 4b). In 2018–2019, meanwhile, group 2 reaches very high counts earlier than group 3, though the curves are quite similar in their shapes (Figure 4c). This latter difference is clear when observing the centroids that resulted selected. Centroids of clusters 1, 2 and 3 for each period studied are presented in Figure S3 in Supplementary Materials.

Comparisons among clusterings for the different time periods showed that the most similar groups are those from the full series and season 2018–2019 according to several dissimilarity measures (see Table S3 in Supplementary Materials). Indeed, some houses are grouped differently in different years. Figure S4 in Supplementary Materials shows the flow among groups in different periods.

**Figure 5:**
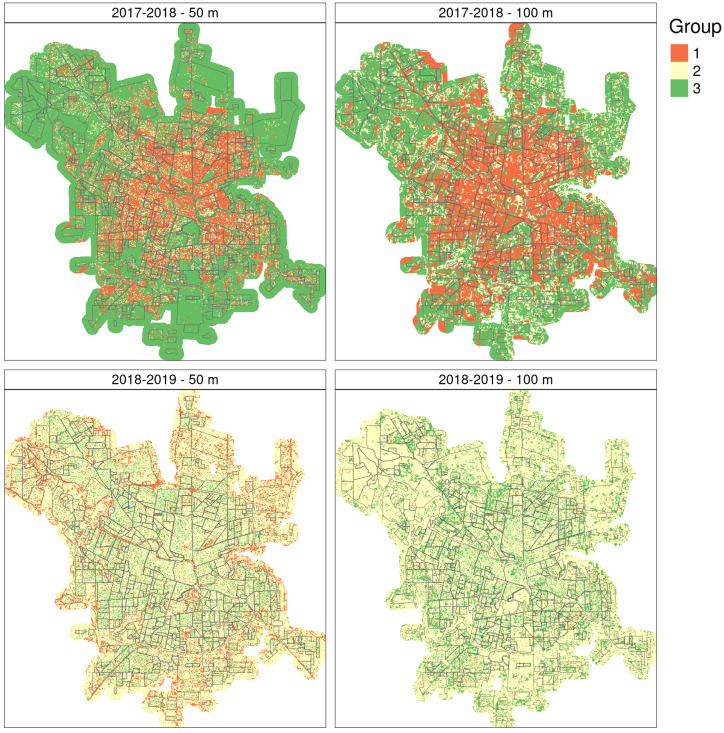
Distribution of temporal clusters as predicted by random forest models for 2017–2018 and 2018–2019 using remote sensing variables taken at ≈50- and 100-m radii. City of Córdoba, Argentina.

**Figure 6:**
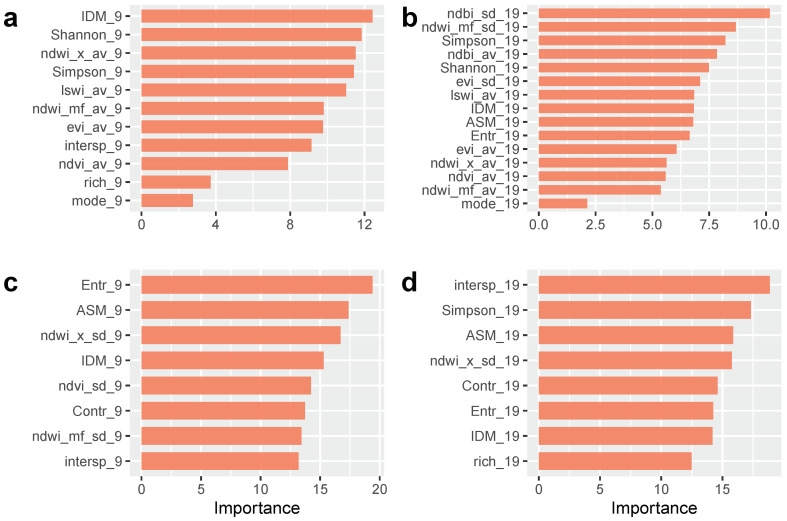
Variable importance from random forest classifications. (**a**) 2017–2018, 50-m radius, (**b**) 2017–2018, 100-m radius, (**c**) 2018–2019, 50-m radius and (**d**) 2018–2019, 100-m radius. References: Sub-indices 9 and 19 represent window sizes in Sentinel 2 pixels, i.e., ≈50 and 100 m radii; av, average; sd, standard deviation; ASM, angular second moment; Contr, contrast; Entr, entropy; IDM, inverse difference moment; intersp, interspertion; EVI, Enhanced Vegetation Index; LSWI, Land Surface Water Index; mode, most common land cover class; NDBI, Normalized Difference Built-up Index; NDVI, Normalized Difference Vegetation Index; NDWI_x, Normalized Difference Water Index by Xu; NDWI_mf, Normalized Difference Water Index by McFeters; Shannon, Shannon diversity index; Simpson, Simpson diversity index; rich, richness—number of different land cover classes.

To try to understand if cluster type was related to any particular environmental condition surrounding ovitraps, we ran random forest classifications with the variables derived from Sentinel 2 image analysis and predicted the results over space (Figure 5). In general, results for 2018–2019 were better than those for 2017–2018 both in terms of overall accuracy (Table 4) and number of variables selected (Figure 6). Diversity indices such as Shannon and Simpson were among the five most important predictors for 2017–2018 models. On the other hand, the texture measure Angular Second Moment (ASM) and NDWI standard deviation were among the most important predictors in models for 2018–2019. The most important variables in the RF model with the highest overall accuracy (Table 4, Figure 6c) included several texture measures, such as entropy, ASM, contrast and inverse difference moment (IDM), and the standard deviation of vegetation and water indices. Figure S5 in Supplementary Materials shows the distribution of the five most important variables in the models fitted with 50-m radius buffer areas in 2017–2018 and 2018–2019 with regards to clustering groups.

**Figure 7:**
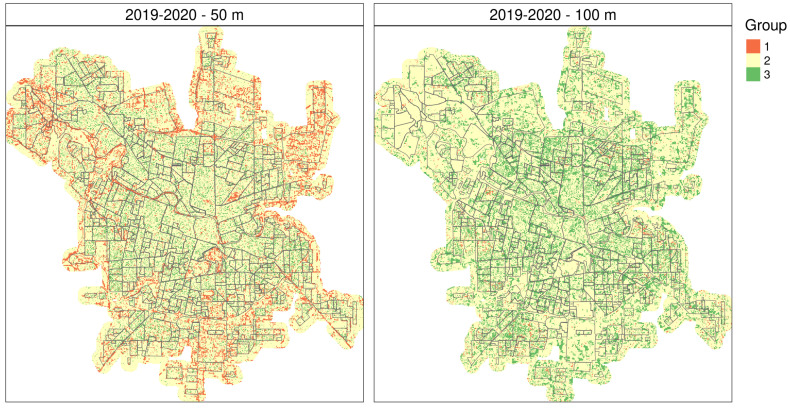
Distribution of temporal clusters for 2019–2020 based on the random forest models for 2018–2019 using remote sensing variables taken at 50- and 100-m radii. City of Córdoba, Argentina.

Since the season 2019–2020 was rather similar to 2018–2019 in terms of weather and environmental data (see Figures S6 and S7 in Supplementary Materials), we used 2018–2019 RF model to predict oviposition temporal pattern over remotely sensed data for 2019–2020, the dengue outbreak season (Figure 7). Since the RF model trained with 50-m radius buffer sizes had a higher training and testing OA and seems to relate better to the spatial configuration of the city too, we used its prediction over space to draw further inferences. The spatial predictions for season 2019–2020, similar to that of 2018–2019, showed a matrix mainly composed of the second temporal pattern, with group 3 mostly within the urban fabric and group 1 in the outskirts or border areas of the city as well as green areas within the city like river banks and the park (Figure 4c and Figure 7 for reference regarding the temporal patterns).

**Figure 8:**
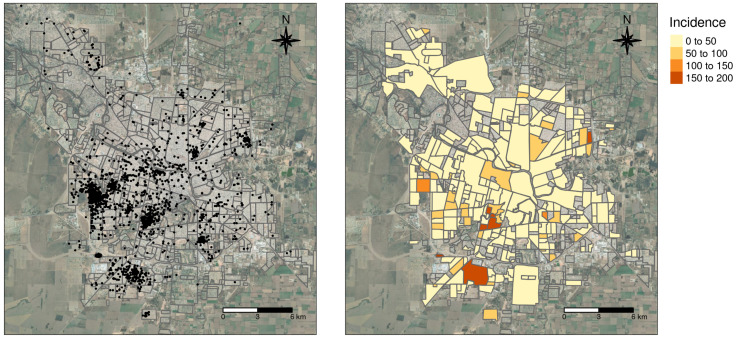
Distribution of autochthonous dengue cases (**left**) and incidence in 10,000 people by neighborhood (**right**) during 2019–2020 season in the city of Córdoba, Argentina.

**Figure 9:**
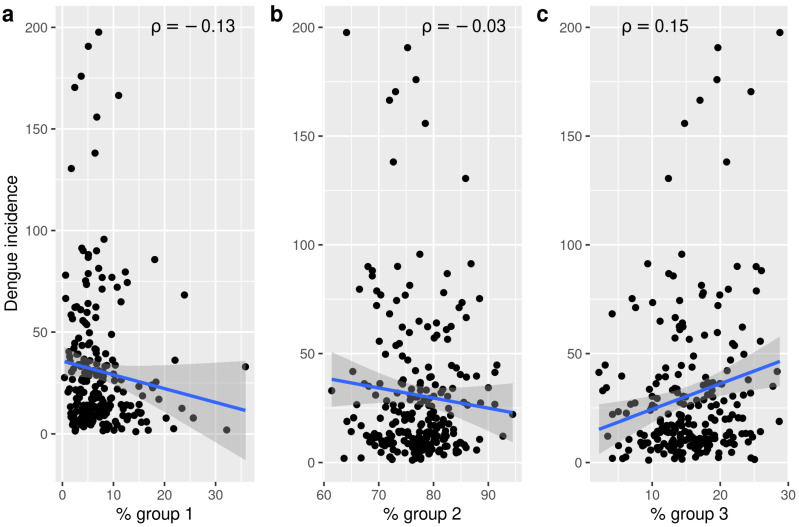
Dengue incidence (number of cases per 10,000 inhabitants) as a function of the percentage of pixels of different temporal patterns per neighbourhood. (**a**) Percentage of the neighbourhood covered by temporal pattern 1; (**b**) Percentage of the neighbourhood covered by temporal pattern 2 and; (**c**) Percentage of the neighbourhood covered by temporal pattern 3.

The distribution of dengue autochthonous cases and incidence in the city of Córdoba is shown in Figure 8. Though cases were recorded all over the urban area, there is a clear concentration in the south-west quadrant with some hotspots towards the east, which is also reflected in the raw incidences. In order to relate dengue occurrence to oviposition temporal patterns for the season 2019–2020, we estimated dengue incidence over 10,000 people and we obtained the percentage of pixels belonging to different temporal patterns in each neighborhood. The Spearman correlations among dengue incidences on a neighborhood basis and the proportion of pixels representing the three different temporal patterns found did not show tight associations (Figure 9). Indeed, only the temporal pattern 3 (see Figure 4c) appeared weakly but positively and significantly related to dengue incidence (ρ = 0.15). Higher proportions of this pattern might eventually imply higher dengue incidence. The other temporal patterns, namely 1 and 2 (Figure 4c), showed negative and barely negative but non-significant correlation with dengue incidences (ρ = −0.13 and −0.03, respectively). In any case, the second temporal pattern is the most commonly found and showed an earlier peak with higher mean egg counts.

## 4. Discussion

This contribution presents a novel approach that combines time series clustering of vector data, remote sensing data and, machine learning to relate oviposition temporal patterns with occurrence of dengue cases. For the different time periods studied, we found three groups of temporal patterns that showed association with environmental features derived from remote sensing data such as land cover diversity, variability in vegetation and water/humidity indices and, heterogeneity as measured by texture estimated over buffer areas of 50- and 100-m radii surrounding ovitraps. Dengue incidence on a neighborhood basis showed however weak association with the percentage of pixels belonging to different temporal patterns.

Consistently with several other studies, we found that oviposition was highly variable though a seasonal pattern was still evident in all cases even with marked differences among periods regarding egg counts and length of seasons [41,42,43,44]. Within this variability, our time series clustering approach, allowed us to identify 3 different temporal patterns over the city in all 3 periods considered (full period, 2017–2018 and 2018–2019). Most algorithms, however, identified one large group and two smaller ones, similarly to the preliminary results presented in [32].

Comparing clusters’ features among seasons (2017–2018 and 2018–2019), we observed that the main differences laid in length of the mosquito season, given by a 20–30 days earlier onset in 2018–2019, and mean egg counts that was two to three times higher in 2018–2019. In terms of time series curve shapes, both seasons displayed a less abundant temporal pattern of low activity and no marked peak (group 2 and group 1, in 2017–2018 and 2018–2019, respectively), and groups with higher egg counts that mainly differ in peak timing, length and height. These latter patterns deserve some attention since they might indicate higher risk if we consider that more oviposition might imply a higher biting rate [45]. The temporal patterns named group 1 in 2017–2018 and group 2 in 2018–2019 are the most common patterns (i.e., higher frequency) and given their characteristics would represent the riskiest conditions in their respective seasons. Indeed, 2018–2019 group 2 implies 2–3 times more oviposition that group 1 in 2017–2018. Group 1 for 2017–2018 displays the highest peak, with oviposition increasing early and fast and the longest period with high egg counts. Similarly, group 2 for 2018–2019 denotes the highest egg counts and the steepest slope (especially evident from Figure S3 in Supplementary Materials). In both seasons, there is a third group which represented a sort of intermediate state, but still more similar to riskier patterns and with similar egg counts. Importantly, the percentage cover of this pattern was the only one that showed a positive relationship with dengue incidence by neighborhood. The temporal groups might then provide guidance on where and when to prioritize epidemiological surveillance actions and promotional campaigns to eliminate mosquito breeding sites.

The spatial distribution of temporal patterns over the city was also variable, with all 3 patterns appearing in different areas of the city. This suggests that they might indeed be explained by local factors, i.e., the *landscape* around the ovitrap. Notably, despite the interannual difference in terms of oviposition activity, the predictions denote a spatial dominance of the temporal patterns representing potentially higher or intermediate risk conditions, especially in 2018–2019 when temporal pattern 2 covers ≈ 75% of the city. In 2017–2018, group 1 is mostly within the city and covers between 22 and 34% of the city. Meanwhile, the temporal pattern representing lower oviposition activity appears either scattered over the city in 2017–2018 or mostly associated to the outskirts and greener areas in 2018–2019 (Figure 5). The spatial predictions for season 2019–2020 showed a spatial distribution of temporal patterns similar to 2018–2019, most likely because of similar weather and environmental conditions during both periods (Figure 7). The dominance of what we have identified as the *riskiest* pattern is indeed more widespread when weather conditions are favorable as in 2018–2019 and 2019–2020, posing a challenge to health authorities in terms of surveillance and breeding sites elimination. This might also imply a much higher risk of dengue outbreak if there are imported cases to trigger virus circulation.

Several studies have attempted to uncover the landscape variables determining breeding hotspots within cities by means of remote sensing data of different spatial resolution. Some have found that heterogeneity and vegetated/urbanized cover are highly relevant to explain *Ae. aegypti* infestation levels [18,19,20,46,47,48]. Importantly, it appears that high environmental variability and heterogeneity in the surroundings of ovitraps might be good predictors of suitability. This can be related to the possibility of finding more potential breeding sites options for females to lay eggs, more places to hide and rest and/or more biting opportunities. Indeed, the best results in terms of train and test overall accuracy, pointed to texture measures and variability in vegetation and water/humidity indices in a radius of 50 m as the most important predictors of oviposition temporal patterns in 2018–2019. In fact, the surroundings of ovitraps belonging to groups 2 and 3 in 2018–2019 were characterized by higher entropy values implying more complex variability, lower IDM and ASM that stand for homogeneity and uniformity, respectively and, higher standard deviation in water and vegetation indices (see Figure S5). Furthermore, results for 2017–2018 season pointed to several diversity measures and average conditions in vegetation and water/humidity indices but OA were much lower, especially for the testing set.

Both in terms of weather and environment, seasons 2018–2019 and 2019–2020 were similar; hence, we used 2018–2019 models to predict oviposition temporal patterns for the season of dengue outbreak. Indeed, predictions were very similar. The relationship among dengue incidence and percentage of different temporal patterns per neighborhood resulted inconclusive. There is however a weak and positive association of incidence and proportion of the 3rd oviposition temporal pattern that would require further refinement with other modeling approaches that were out of the scope of this contribution. Indeed, previous studies reviewing the relationship between dengue and vector indices report highly variable results [12,49]. Still, Cromwell et al. found that entomological information from temporal studies was related to dengue seroconversion while data from cross-sectional studies were not. Hence, sustaining vector surveillance in space and time will likely be beneficial in understanding the vector-disease relationship.

The approach we propose here is aimed at contributing to the understanding of mosquito activity patterns within urban areas in contrast to other studies that aggregate both mosquito and environmental data and consider cities as homogeneous packs and predict mean oviposition or abundance values [22,23,43,50,51]. In this regard, the only studies assuming differences within cities regarding different temporal patterns are [24,32]. The former, however, does not use time series clustering but a regular k-means in order to group data before training deep learning LSTM networks and forecasting adult mosquito abundance by group. Meanwhile, in our previous contribution [32], we raised some potential pitfalls that have been addressed to some extent here. In fact, we used different buffer sizes, we used aggregated remote sensing data instead of just one scene and we performed the analysis with effectively two years of data instead of only one. Furthermore, given the occurrence of a dengue outbreak, we were able to somehow relate the oviposition pattern distribution with dengue cases occurrence, though we were not able to assess any thresholds. This would still require further investigation because given the COVID-19 pandemic and suppression of many activities, we could not have simultaneous oviposition and dengue data. In any case, a similar analysis in places with usual circulation of dengue virus would be relevant as to understand if there are thresholds that might indicate risk and how different groups identified by clustering relate to risk.

It is important to remark that there might be some limitations to our approach that could influence the results. Indeed, since the first step in our workflow can be considered a data driven analysis, we do not have ground truth data to validate the groups obtained. Moreover, because of the limited amount of time series per cluster we used up-sampling before training random forest classification in order to have somehow balanced groups. This might have influenced model’s fit as seen in the comparison among training and test accuracy. Similarly, the lower proportion of surface predicted for the lowest activity groups (2 in 2017–2018 and 1 in 2018–2019) might be related to the limited number of time series that were clustered together in these groups. In this sense, the distribution of monitoring ovitraps over the city of Córdoba has changed for season 2020–2021, and those data could be used in further analysis as an independent set to assess predictions. Importantly, because our study focuses on land cover influences, weather variables such as temperature and precipitation were not included. We do not discard, however, that micro-climate differences between ovitraps locations could be relevant to explain the variations in temporal patterns. However, while we might obtain land surface temperature from Landsat at 30 m every 16 days at best, there is not yet a ready-to-use gridded precipitation product with such resolution, and still, better temporal revisit would be needed anyway. Furthermore, our analysis as well as most other studies, cannot account for human behavior in the surrounding of monitoring stations, i.e., plant watering/container re-filling, availability of other containers inside and outside the dwelling, breeding sites removal, pets tipping over containers, etc. However, we pledge that to be able to intervene in a timely manner, we need to study within city variations and micro-habitat mosquito preferences, especially in large cities such as Córdoba and in the face of limited resources. In an attempt to go into this direction, we are currently analyzing the environmental variability at very high spatial resolution (50 cm) in order to suggest a distribution of trapping stations that accounts for such within city variations.

## 5. Conclusions

In the present work, with the ultimate goal of predicting risk in places with no mosquito field data, we have explored a novel approach that combines time series clustering of weekly ovitrap records, satellite imagery and machine learning classification tools to assess the association of temporal patterns with environmental variables and finally test the relationship between oviposition and dengue incidence.

The temporal patterns found denoted differences regarding egg counts, peak existence, timing, length and height. The temporal clusters showed association with environmental features, such as land cover diversity, variability in vegetation and water/humidity and texture indices estimated from remote sensing data. Dengue incidence on a neighborhood basis showed a weak but positive association with the percentage of pixels belonging to a temporal pattern occurring only within the urban area and with the highest average of maximum egg counts.

The understanding of the spatial distribution of temporal patterns and their environmental determinants becomes highly relevant to guide the timing and allocation of interventions, especially if we consider that more oviposition might imply higher female biting rates in previous days within a buffer area of a certain ovitrap. Further investigation is still needed though to incorporate other determinants (especially related to human behavior) not considered here. In any case, sustaining long term vector surveillance programs is key for a better understanding of the vector-environment-disease system.

## Figures and Tables

**Table 1:** Combinations of distances and centroid extraction methods tested and compared. References: DTW, Dynamic Time Warping; DTW_LB, Dynamic Time Warping Lower Bounds; DBA, DTW Barycenter Averaging; PAM, Partition Around Medoids; SBD, Shape Based Distance.

Distance	Centroid	Pre-Processing	Configurations
DTW	DBA	Normalization	1600
DTW_LB	DBA	Normalization	1600
DTW	PAM	Normalization	1600
DTW_LB	PAM	Normalization	1600
SBD	PAM	Normalization	160
SBD	Shape extraction	Normalization	160

**Table 2:** Results of the best clustering configurations per type of algorithm. References: rep, repetition, k, number of clusters, n, number of time series in each cluster, DTW, Dynamic Time Warping; PAM, Partition Around Medoids; SBD, shape based distance, Sil, Silhouette; DB, Davies–Bouldin; DB*, Modified Davies–Bouldin; D, Dunn; COP, COP index.

Series	Rep	k	n	Dist	Cent	Dist Wind Size	Norm Dist	Cent Wind Size	Norm Cent	Znorm Cent	Sil	D	COP	DB	DB*	Votes
Full series	10	3	89,44,10	dtw_basic	dba	4	L2	4	L2		0.095	0.311	2.026	0.581	0.581	1
	10	3	89,44,10	dtw_lb	dba	4	L2	4	L2		0.158	0.076	1.697	0.413	0.404	0
	2	3	16,26,101	dtw_basic	pam	5	L2				0.100	0.315	1.867	0.739	0.702	**3**
	7	3	110,9,24	dtw_lb	pam	3	L2				0.192	0.099	1.700	0.445	0.436	1
	7	3	6,111,26	sbd	shape					TRUE	0.181	0.123	1.700	0.575	0.562	0
	1	3	25,12,106	sbd	pam						0.098	0.203	2.023	0.537	0.489	0
2017–2018	10	4	30,71,14,28	dtw_basic	dba	4	L2	4	L2		0.058	0.233	1.889	0.615	0.612	1
	1	3	54,37,52	dtw_lb	dba	2	L2	2	L2		0.119	0.098	1.602	0.454	0.454	1
	7	3	70,30,43	dtw_basic	pam	5	L1				0.049	0.234	1.551	0.711	0.688	**2**
	5	5	44,49,29,10,11	dtw_lb	pam	1	L2				0.106	0.167	1.423	0.534	0.517	0
	8	3	48,30,65	sbd	shape					TRUE	0.097	0.191	2.422	0.665	0.627	1
	10	3	41,44,58	sbd	pam						0.076	0.112	1.958	0.507	0.492	0
2018–2019	8	3	10,89,44	dtw_basic	dba	4	L2	4	L2		0.140	0.301	1.985	0.692	0.663	1
	3	4	37,42,13,51	dtw_lb	dba	3	L2	3	L2		0.132	0.053	1.510	0.429	0.423	0
	10	4	24,21,77,21	dtw_basic	pam	5	L2				0.084	0.295	1.883	0.703	0.679	1
	7	3	25,39,79	dtw_lb	pam	5	L1				0.254	0.014	0.586	0.120	0.117	1
	6	3	10,88,45	sbd	shape					TRUE	0.111	0.136	2.667	0.702	0.654	**2**
	4	3	37,37,69	sbd	pam						0.075	0.082	2.307	0.639	0.627	0

**Table 3:** Median start and end date of oviposition in the best clustering configurations for 2017–2018 and 2018–2019. References: n, number of time series in each cluster; MSD, median start date; MED, median end date.

Series	Cluster	n	MSD	MED	Duration (Days)	Max Eggs	Mean (Max Eggs)	Date of Max (Eggs)
2017–2018	1	70	20 November 2017	7 May 2018	168	205	73	1 February 2018
	2	30	4 December 2017	7 May 2018	154	155	55	15 January 2018
	3	43	27 November 2017	30 April 2018	154	196	74	29 January 2018
2018–2019	1	10	25 October 2018	29 April 2019	186	274	127	11 February 2019
	2	88	5 November 2018	29 April 2019	175	594	190	21 January 2019
	3	45	29 October 2018	29 April 2019	182	615	206	4 February 2019

**Table 4:** Train and test overall accuracy for random forest classifications in different periods and using buffers of 50- and 100-m radius.

		2017–2018	2018–2019
Train	50 m	0.733 (0.083)	0.822 (0.051)
	100 m	0.730 (0.080)	0.778 (0.090)
Test	50 m	0.381	0.524
	100 m	0.476	0.500

## Data Availability

The data are available upon a direct request to the Córdoba province Health Ministry.

## Supplementary Materials

The following are available online at https://www.mdpi.com/article/10.3390/insects12100919/s1: Table S1: List of Sentinel 2 scenes used. Table S2: Variables derived from satellite image analysis used for modeling the cluster type in Córdoba (Argentina). Table S3: Clustering comparisons by means of dissimilarity measures. Figure S1: Natural color Sentinel 2 band combinations for years 2017–2018 (A), 2018–2019 (B) and 2019–2020 (C) for Córdoba city and surroundings (RBG 4,3,2). Figure S2: Unsupervised classification for years 2017–2018, 2018–2019 and 2019–2020 for Córdoba city and surroundings. Figure S3: Centroid time series of the clusters obtained for (a) the full series, (b) 2017–2018 and (c) 2018–2019. Values are normalized. Figure S4: Clusters flow among different periods. Figure S5: Comparison clusters in terms of the five most important variables for models fitted with 50-m radius buffer areas in 2017–2018 (a, c, e, g, i) and 2018–2019 (b, d, f, h, j). Figure S6: Time series of precipitation, LST day and night, EVI, NDVI and NDWI for the period 2017–2020 in Córdoba city, Argentina. Figure S7: Comparison of precipitation, LST day and night, EVI, NDVI and NDWI for the core mosquito season (November to March) in the periods 2017–2018 and 2018–2019 and 2019–2020 in Córdoba city, Argentina.

## References

[1] Vezzani D., Carbajo A.E. (2008). Aedes aegypti, Aedes albopictus, and dengue in Argentina: Current knowledge and future directions. Memórias do Instituto Oswaldo Cruz.

[2] Liu-Helmersson J., Rocklov J., Sewe M., Brannstrom A. (2019). Climate change may enable *Aedes aegypti* infestation in major European cities by 2100. Environ. Res..

[3] Rubio A., Cardo M.V., Vezzani D., Carbajo A.E. (2020). Aedes aegypti spreading in South America: New coldest and southernmost records. Memórias do Instituto Oswaldo Cruz.

[4] Powell J.R., Tabachnick W.J. (2013). History of domestication and spread of *Aedes aegypti*—A Review. Memórias do Instituto Oswaldo Cruz.

[5] Wilke A.B.B., Chase C., Vasquez C., Carvajal A., Medina J., Petrie W.D., Beier J.C. (2019). Urbanization creates diverse aquatic habitats for immature mosquitoes in urban areas. Sci. Rep..

[6] Stanaway J.D., Shepard D.S., Undurraga E.A., Halasa Y.A., Coffeng L.E., Brady O.J., Hay S.I., Bedi N., Bensenor I.M., Castañeda-Orjuela C.A. (2016). The global burden of dengue: An analysis from the Global Burden of Disease Study 2013. Lancet Infect. Dis..

[7] Seijo A., Romer Y., Espinosa M., Monroig J., Giamperetti S., Ameri D., Antonelli L.G. (2009). Outbreak of indigenous dengue in the Buenos Aires Metropolitan Area. Experience of the F. J. Muñíz Hospital. Medicina.

[8] Estallo E.L., Carbajo A.E., Grech M.G., Frías-Céspedes M., López L., Lanfri M.A., Ludueña-Almeida F.F., Almirón W.R. (2014). Spatio-temporal dynamics of dengue 2009 outbreak in Córdoba City, Argentina. Acta Trop..

[9] Rotela C., Lopez L., Céspedes M.F., Barbas G., Lighezzolo A., Porcasi X., Lanfri M.A., Scavuzzo C.M., Gorla D.E. (2017). Analytical report of the 2016 dengue outbreak in Córdoba city, Argentina. Geospat. Health.

[10] Robert M.A., Tinunin D.T., Benitez E.M., Ludueña-Almeida F.F., Romero M., Stewart-Ibarra A.M., Estallo E.L. (2019). Arbovirus emergence in the temperate city of Córdoba, Argentina, 2009–2018. Sci. Data.

[11] Ministerio de Salud de la Nación (2020). Boletín Integrado de Vigilancia.

[12] Bowman L.R., Donegan S., McCall P.J. (2016). Is Dengue Vector Control Deficient in Effectiveness or Evidence? Systematic Review and Meta-analysis. PLoS Neglected Trop. Dis..

[13] Getis A., Morrison A.C., Gray K., Scott T.W. (2003). Characteristics of the spatial pattern of the Dengue vector, *Aedes aegypti*, in Iquitos, Perú. Am. J. Trop. Med. Hyg..

[14] Sallam M.F., Fizer C., Pilant A.N., Whung P.Y. (2017). Systematic Review: Land Cover, Meteorological, and Socioeconomic Determinants of Aedes Mosquito Habitat for Risk Mapping. Int. J. Environ. Res. Public Health.

[15] Marti R., Li Z., Catry T., Roux E., Mangeas M., Handschumacher P., Gaudart J., Tran A., Demagistri L., Faure J.F. (2020). A Mapping Review on Urban Landscape Factors of Dengue Retrieved from Earth Observation Data, GIS Techniques, and Survey Questionnaires. Remote Sens..

[16] Porcasi X., Rotela C.H., Introini M.V., Frutos N., Lanfri S., Peralta G., Elia E.A.D., Lanfri M.A., Scavuzzo C.M. (2012). An operative dengue risk stratification system in Argentina based on geospatial technology. Geospat. Health.

[17] Estallo E.L., Sangermano F., Grech M., Ludueña-Almeida F., Frías-Cespedes M., Ainete M., Almirón W., Livdahl T. (2018). Modelling the distribution of the vector *Aedes aegypti* in a central Argentine city: Modelling Aedes aegypti distribution. Med. Vet. Entomol..

[18] Benitez E.M., Ludueña-Almeida F., Frías-Céspedes M., Almirón W.R., Estallo E.L. (2020). Could land cover influence *Aedes aegypti* mosquito populations?. Med. Vet. Entomol..

[19] Lorenz C., Castro M.C., Trindade P.M.P., Nogueira M.L., de Oliveira Lage M., Quintanilha J.A., Parra M.C., Dibo M.R., Fávaro E.A., Guirado M.M. (2020). Predicting Aedes aegypti infestation using landscape and thermal features. Sci. Rep..

[20] Andreo V., Cuervo P.F., Porcasi X., Lopez L., Guzman C., Scavuzzo C.M. (2021). Towards a workflow for operational mapping of Aedes aegypti at urban scale based on remote sensing. Remote Sens. Appl. Soc. Environ..

[21] Aguirre E., Andreo V., Porcasi X., Lopez L., Guzman C., González P., Scavuzzo C.M. (2021). Implementation of a proactive system to monitor Aedes aegypti populations using open access historical and forecasted meteorological data. Ecol. Inform..

[22] Estallo E.L., Benitez E.M., Lanfri M.A., Scavuzzo C.M., Almirón W.R. (2016). MODIS Environmental Data to Assess Chikungunya, Dengue, and Zika Diseases Through Aedes (Stegomia) aegypti Oviposition Activity Estimation. IEEE J. Sel. Top. Appl. Earth Obs. Remote Sens..

[23] Scavuzzo J.M., Trucco F., Espinosa M., Tauro C.B., Abril M., Scavuzzo C.M., Frery A.C. (2018). Modeling Dengue vector population using remotely sensed data and machine learning. Acta Trop..

[24] Mudele O., Frery A.C., Zanandrez L.F.R., Eiras A.E., Gamba P. (2021). Dengue Vector Population Forecasting Using Multisource Earth Observation Products and Recurrent Neural Networks. IEEE J. Sel. Top. Appl. Earth Obs. Remote Sens..

[25] Andreo V., Izquierdo-Verdiguier E., Zurita-Milla R., Rosa R., Rizzoli A., Papa A. Identifying Favorable Spatio-Temporal Conditions for West Nile Virus Outbreaks by Co-Clustering of Modis LST Indices Time Series. Proceedings of the IGARSS 2018—2018 IEEE International Geoscience and Remote Sensing Symposium.

[26] Albrieu-Llinás G., Espinosa M.O., Quaglia A., Abril M., Scavuzzo C.M. (2018). Urban environmental clustering to assess the spatial dynamics of *Aedes aegypti* breeding sites. Geospat. Health.

[27] Aghabozorgi S., Seyed Shirkhorshidi A., Ying Wah T. (2015). Time-series clustering—A decade review. Inf. Syst..

[28] Franses P.H., Wiemann T. (2020). Intertemporal Similarity of Economic Time Series: An Application of Dynamic Time Warping. Comput. Econ..

[29] Belgiu M., Csillik O. (2017). Sentinel-2 cropland mapping using pixel-based and object-based time-weighted dynamic time warping analysis. Remote Sens. Environ..

[30] Naranjo-Fernández N., Guardiola-Albert C., Aguilera H., Serrano-Hidalgo C., Montero-González E. (2020). Clustering Groundwater Level Time Series of the Exploited Almonte-Marismas Aquifer in Southwest Spain. Water.

[31] Zhang Y., Hepner G.F. (2017). The Dynamic-Time-Warping-based k-means++ clustering and its application in phenoregion delineation. Int. J. Remote Sens..

[32] Andreo V., Porcasi X., Rodriguez C., Lopez L., Guzman C., Scavuzzo C.M. Time Series Clustering Applied to Eco-Epidemiology: The case of *Aedes aegypti* in Córdoba, Argentina. Proceedings of the 2019 XVIII Workshop on Information Processing and Control (RPIC).

[33] INDEC (Instituto Nacional de Estadística y Censos) (2010). National Population, Homes and Houses Census.

[34] Espinosa M., Alvarez Di Fino E.M., Abril M., Lanfri M., Periago M.V., Scavuzzo C.M. (2018). Operational satellite-based temporal modelling of Aedes population in Argentina. Geospat. Health.

[35] GRASS Development Team (2019). Geographic Resources Analysis Support System (GRASS GIS) Software.

[36] Arbelaitz O., Gurrutxaga I., Muguerza J., Pérez J.M., Perona I. (2013). An extensive comparative study of cluster validity indices. Pattern Recognit..

[37] Sarda-Espinosa A. (2019). Dtwclust: Time Series Clustering Along with Optimizations for the Dynamic Time Warping Distance.

[38] R Core Team (2020). R: A Language and Environment for Statistical Computing.

[39] Hornik K. (2021). Clue: Cluster Ensembles.

[40] Kuhn M. (2021). Caret: Classification and Regression Training.

[41] Micieli M.V., Campos R.E. (2003). Oviposition activity and seasonal pattern of a population of *Aedes* (*Stegomyia*) *aegypti* (L.) (Diptera: Culicidae) in subtropical Argentina. Memórias do Instituto Oswaldo Cruz.

[42] Estallo E.L., Ludueña-Almeida F.F., Visintin A.M., Scavuzzo C.M., Introini M.V., Zaidenberg M., Almirón W.R. (2011). Prevention of Dengue Outbreaks Through *Aedes aegypti* Oviposition Activity Forecasting Method. Vector-Borne Zoonotic Dis..

[43] German A., Espinosa M.O., Abril M., Scavuzzo C.M. (2018). Exploring satellite based temporal forecast modelling of Aedes aegypti oviposition from an operational perspective. Remote Sens. Appl. Soc. Environ..

[44] Benitez E.M., Estallo E.L., Grech M.G., Frías-Céspedes M., Almirón W.R., Robert M.A., Ludueña-Almeida F.F. (2021). Understanding the role of temporal variation of environmental variables in predicting Aedes aegypti oviposition activity in a temperate region of Argentina. Acta Trop..

[45] Manica M., Rosà R., Torre A.D., Caputo B. (2017). From eggs to bites: Do ovitrap data provide reliable estimates of Aedes albopictus biting females?. PeerJ.

[46] Landau K.I., Leeuwen W.J.D.V. (2012). Fine scale spatial urban land cover factors associated with adult mosquito abundance and risk in Tucson, Arizona. J. Vector Ecol..

[47] Espinosa M.O., Polop F., Rotela C.H., Abril M., Scavuzzo C.M. (2016). Spatial pattern evolution of Aedes aegypti breeding sites in an Argentinean city without a dengue vector control programme. Geospat. Health.

[48] Chen S., Whiteman A., Li A., Rapp T., Delmelle E., Chen G., Brown C.L., Robinson P., Coffman M.J., Janies D. (2019). An operational machine learning approach to predict mosquito abundance based on socioeconomic and landscape patterns. Landsc. Ecol..

[49] Cromwell E.A., Stoddard S.T., Barker C.M., Rie A.V., Messer W.B., Meshnick S.R., Morrison A.C., Scott T.W. (2017). The relationship between entomological indicators of *Aedes aegypti* abundance and dengue virus infection. PLoS Neglected Trop. Dis..

[50] Lee K.Y., Chung N., Hwang S. (2016). Application of an artificial neural network (ANN) model for predicting mosquito abundances in urban areas. Ecol. Inform..

[51] Espinosa M., Weinberg D., Rotela C.H., Polop F., Abril M., Scavuzzo C.M. (2016). Temporal Dynamics and Spatial Patterns of Aedes aegypti Breeding Sites, in the Context of a Dengue Control Program in Tartagal (Salta Province, Argentina). PLoS Neglected Trop. Dis..

