# Supplementary material for: Spatial Distribution of Aedes aegypti Oviposition Temporal Patterns and Their Relationship with Environment and Dengue Incidence
Source: Insects. 2021 Oct 9;12(10):919. doi: 10.3390/insects12100919 (PMC8537924; doi:10.3390/insects12100919)
Supplement: Supplementary file 1 [file insects-12-00919-s001.zip › insects-1342533-supplementary.pdf]

Supplementary Materials: Spatial Distribution of *Aedes aegypti* Oviposition Temporal Patterns and Their Relationship with Environment and Dengue Incidence

Verónica Andreo [1,2,*], Ximena Porcasi [1], Claudio Guzman [3], Laura Lopez [3] and Carlos M. Scavuzzo [1]

Table S1. List of Sentinel 2 scenes used.

Period	Date	Scene name	Cloud %
2017/2018	2017-11-14	S2B_MSIL1C_20171114T142029_N0206_R010_T20JLL_20171114T185810	0.0
	2017-12-14	S2B_MSIL1C_20171214T142029_N0206_R010_T20JLL_20171214T173517	0.0
	2018-01-15	S2A_MSIL1C_20180115T141041_N0206_R110_T20JLL_20180115T173441	0.0
	2018-02-14	S2A_MSIL1C_20180214T141041_N0206_R110_T20JLL_20180214T203900	0.0
	2018-03-21	S2B_MSIL1C_20180321T141039_N0206_R110_T20JLL_20180321T173031	0.0
2018/2019	2018-11-14	S2A_MSIL1C_20181114T142031_N0207_R010_T20JLL_20181114T175244	1.0
	2018-12-14	S2A_MSIL1C_20181214T142031_N0207_R010_T20JLL_20181214T173648	1.0
	2019-01-20	S2A_MSIL1C_20181214T142031_N0207_R010_T20JLL_20181214T173648	5.0
	2019-02-14	S2A_MSIL1C_20181214T142031_N0207_R010_T20JLL_20181214T173648	0.0
	2019-03-24	S2A_MSIL1C_20190324T142041_N0207_R010_T20JLL_20190324T205705	6.0
2019/2020	2019-11-29	S2A_MSIL1C_20190324T142041_N0207_R010_T20JLL_20190324T205705	9.0
	2019-12-09	S2A_MSIL1C_20191209T141731_N0208_R010_T20JLL_20191209T173854	1.0
	2020-01-28	S2A_MSIL1C_20200128T141651_N0208_R010_T20JLL_20200128T173915	0.0
	2020-02-12	S2B_MSIL1C_20200212T141649_N0209_R010_T20JLL_20200212T174151	7.0
	2020-03-20	S2B_MSIL1C_20200320T141049_N0209_R110_T20JLL_20200320T173058	0.0

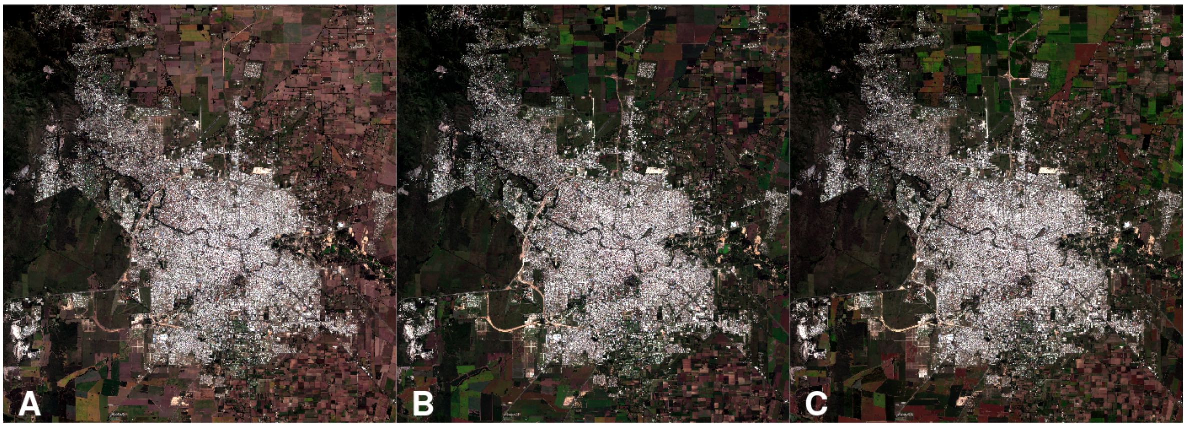

Figure S1. Natural color Sentinel 2 band combinations for years 2017-2018 (A), 2018-2019 (B) and 2019-2020 (C) for Córdoba city and surroundings (RBG 4,3,2).

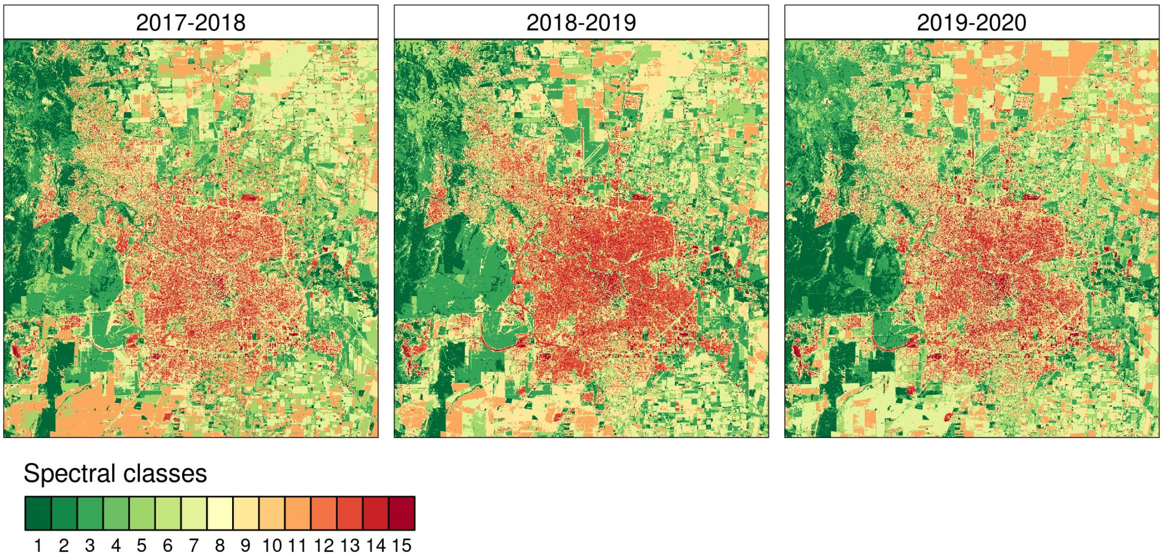

Figure S2. Unsupervised classification for years 2017-2018, 2018-2019 and 2019-2020 for Córdoba city and surroundings.

Table S2. Variables derived from satellite image analysis used for modeling the cluster type in Córdoba (Argentina).

Variable	Description	Example name
EVI Average	Average of Enhanced Vegetation Index (EVI) in an area of 50 or 100 m radio.	evi_av_9
EVI Standard Deviation	Standard deviation of Enhanced Vegetation Index (EVI) in an area of 50 or 100 m radio.	evi_sd_9
LSWI Average	Average of Land Surface Water Index (LSWI) in an area of 50 or 100 m radio.	lswi_av_9
LSWI Standard Deviation	Standard deviation of Land Surface Water Index (LSWI) in an area of 50 or 100 m radio.	lswi_sd_9
NDBI Average	Average of Normalized Difference Built-up Index (NDBI) in an area of 50 or 100 m radio.	ndbi_av_9
NDBI Standard Deviation	Standard deviation of Normalized Difference Built-up Index (NDBI) in an area of 50 or 100 m radio.	ndbi_sd_9
NDVI Average	Average of Normalized Difference Vegetation Index (NDVI) in an area of 50 or 100 m radio.	ndvi_av_9
NDVI Standard Deviation	Standard deviation of Normalized Difference Vegetation Index (NDVI) in an area of 50 or 100 m radio.	ndvi_sd_9
NDWI Average (McFeters)	Average of Normalized Difference Water Index (NDWI) in an area of 50 or 100 m radio.	ndwi_mf_av_9
NDWI Standard Deviation (McFeters)	Standard deviation of Normalized Difference Water Index (NDWI) in an area of 50 or 100 m radio.	ndwi_mf_sd_9
NDWI Average (Xu)	Average of Normalized Difference Water Index (NDWI) in an area of 50 or 100 m radio.	ndwi_x_av_9
NDWI Standard Deviation (Xu)	Standard deviation of Normalized Difference Water Index (NDWI) in an area of 50 or 100 m radio.	ndwi_x_sd_9
Angular Second Moment (ASM)	Angular second moment estimated over a synthetic panchromatic band in an area of 50 or 100 m radio.	ASM_9
Contrast	Contrast estimated over a synthetic panchromatic band in an area of 50 or 100 m radio.	Contr_9
Correlation	Correlation estimated over a synthetic panchromatic band in an area of 50 or 100 m radio.	Corr_9
Entropy	Entropy estimated over a synthetic panchromatic band in an area of 50 or 100 m radio.	Entr_9
Inverse Difference Moment (IDM)	Inverse difference moment estimated over a synthetic panchromatic band in an area of 50 or 100 m radio.	IDM_9
Richness	Number of different land cover classes in an area of 50 or 100 m radio.	rich_9
Interspertion	Proportion or cells belonging to land cover classes different to that of the central pixel in an area of 50 or 100 m radio.	intersp_9
Mode	Most common land cover class in an area of 50 or 100 m radio.	mode_9
Diversity Simpson	Simpson diversity index estimated over land cover classes in an area of 50 or 100 m radio.	Simpson_9
Diversity Shannon	Shannon diversity index estimated over land cover classes in an area of 50 or 100 m radio.	Shannon_9

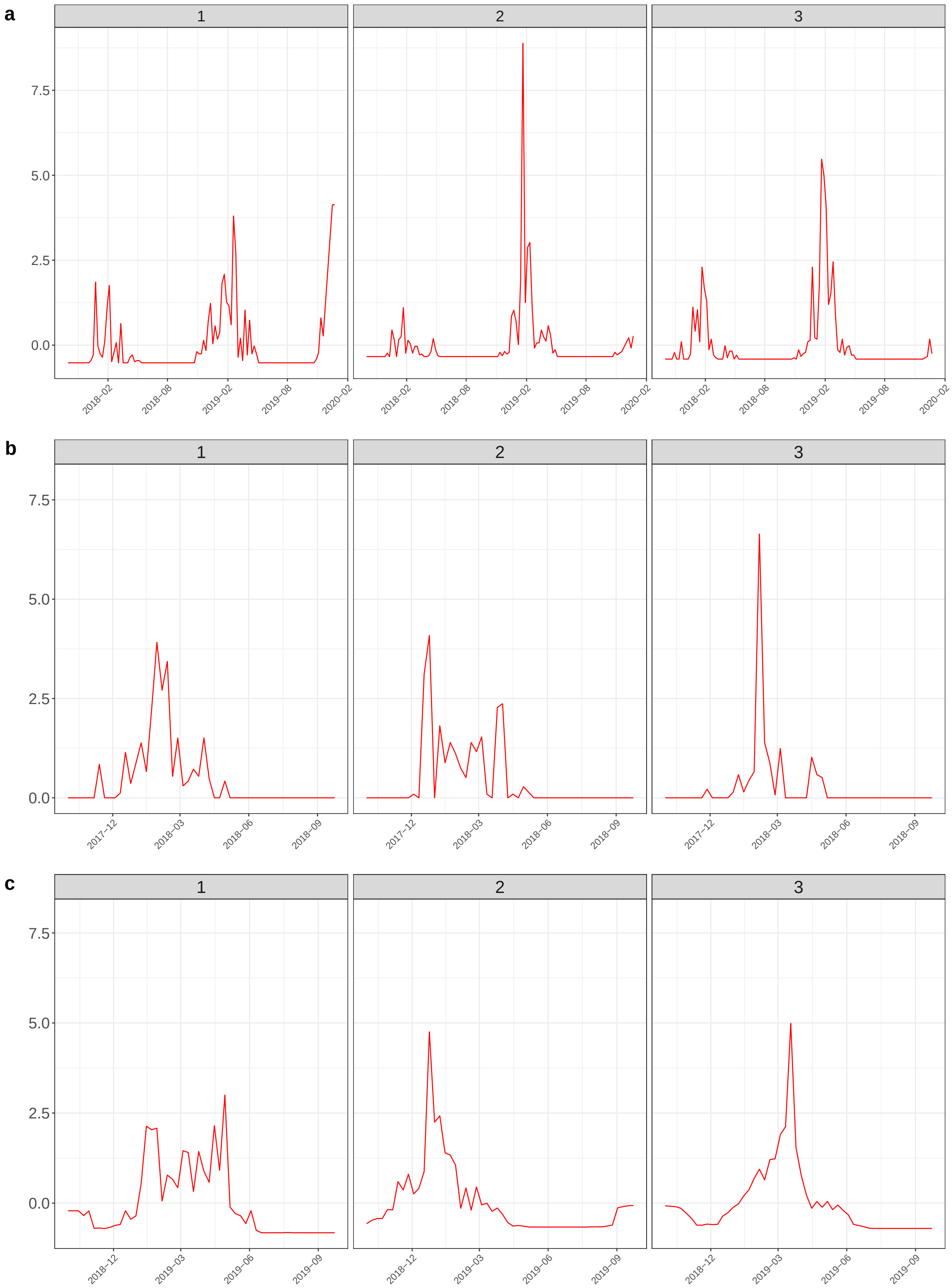

Figure S3. Centroid time series of the clusters obtained for a) the full series, b) 2017-2018 and c) 2018-2019. Values are normalized.

Table S3. Clustering comparisons by means of dissimilarity measures.

	Dissimilarity measure					
comparison	euclidean	manhattan	comemberships	symdiff	Rand	VI
Full-1718	12.649	160	101.094	5110	0.503	1.825
Full-1819	11.662	136	99.94	4994	0.492	1.583
1718-1819	13.191	174	101.232	5124	0.505	1.851

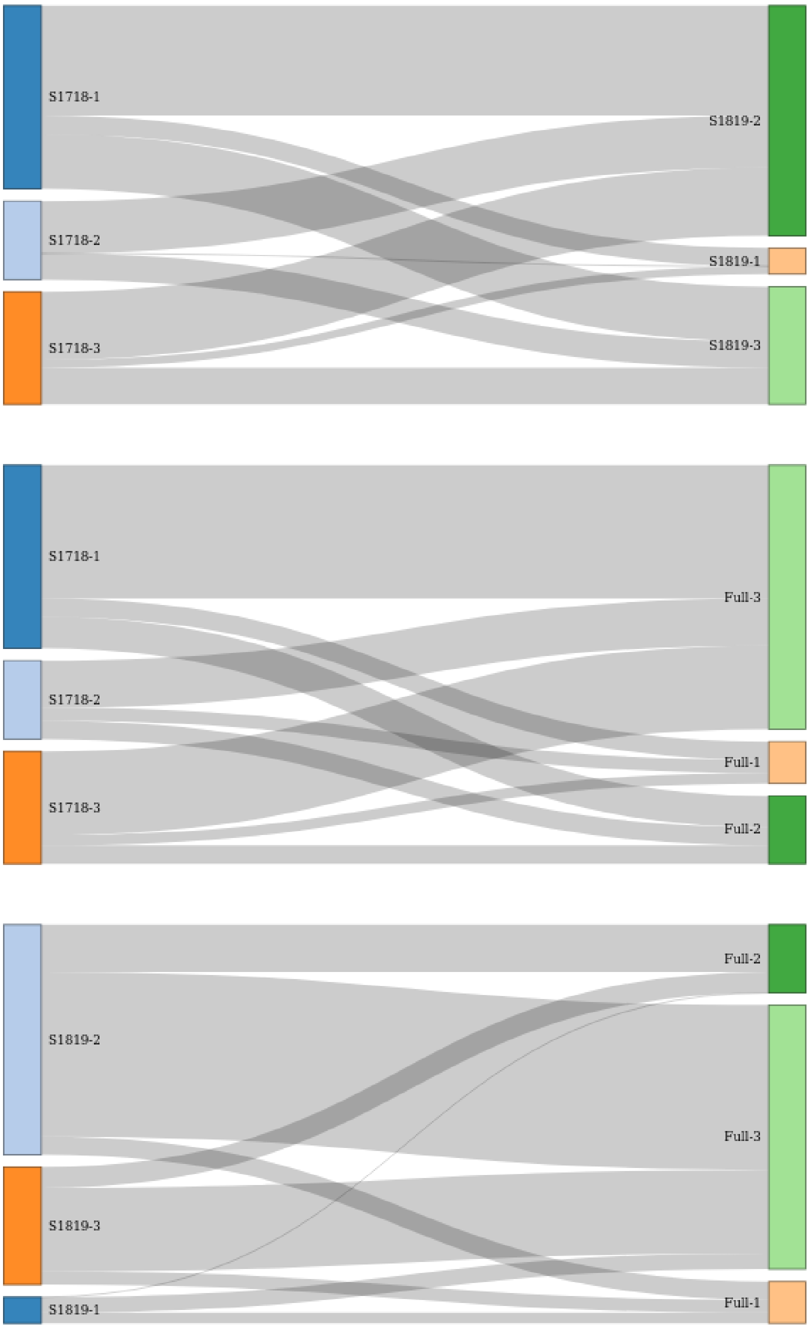

Figure S4. Clusters flow among different periods.

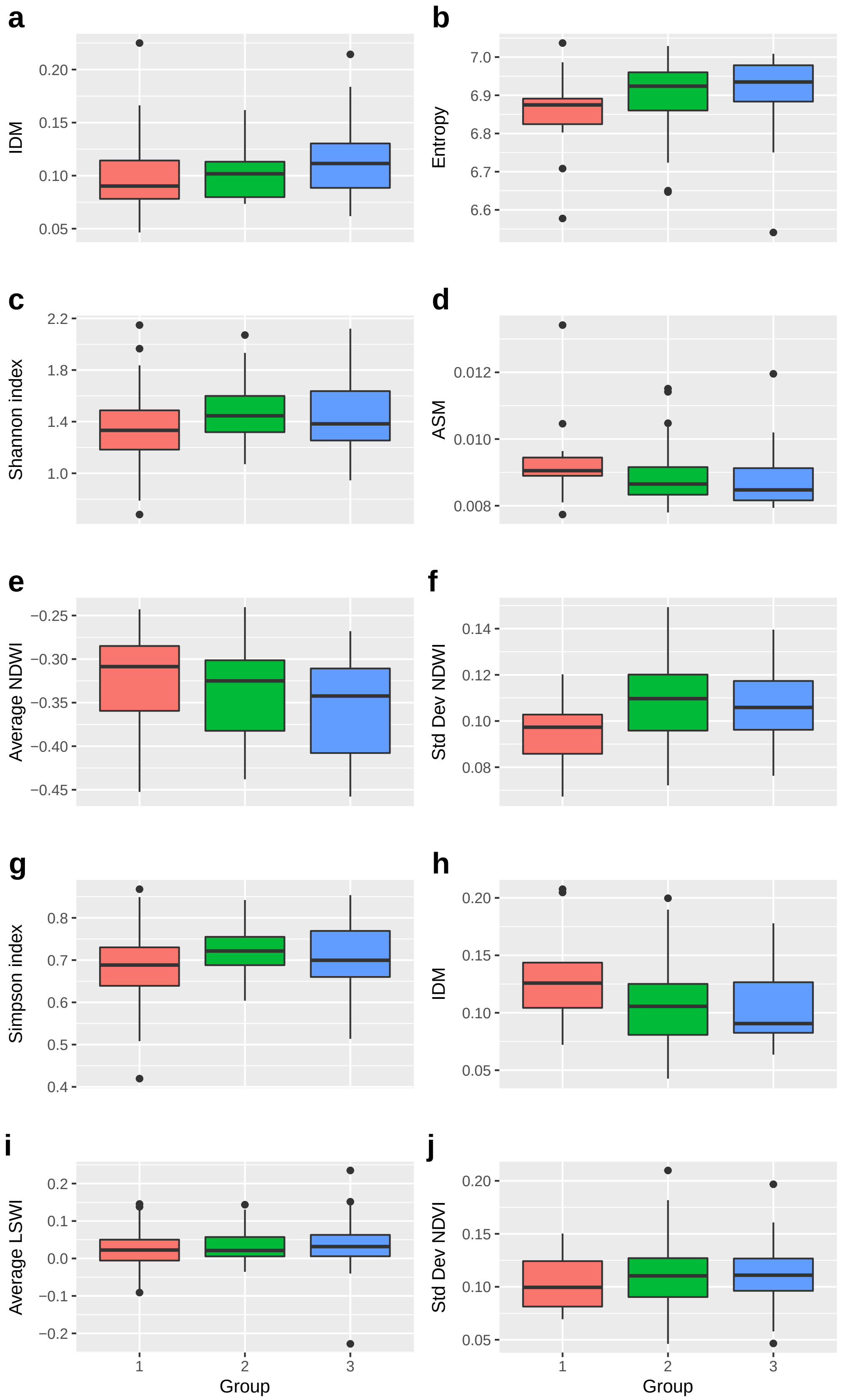

Figure S5. Comparison clusters in terms of the five most important variables for models fitted with 50 m radio buffer areas in 2017-2018 (a, c, e, g, i) and 2018-2019 (b, d, f, h, j). References: IDM, inverse difference moment; ASM, angular second moment; NDWI, normalized difference water index; LSWI, land surface water index; NDVI, normalized difference vegetation index; Std Dev, standard deviation.

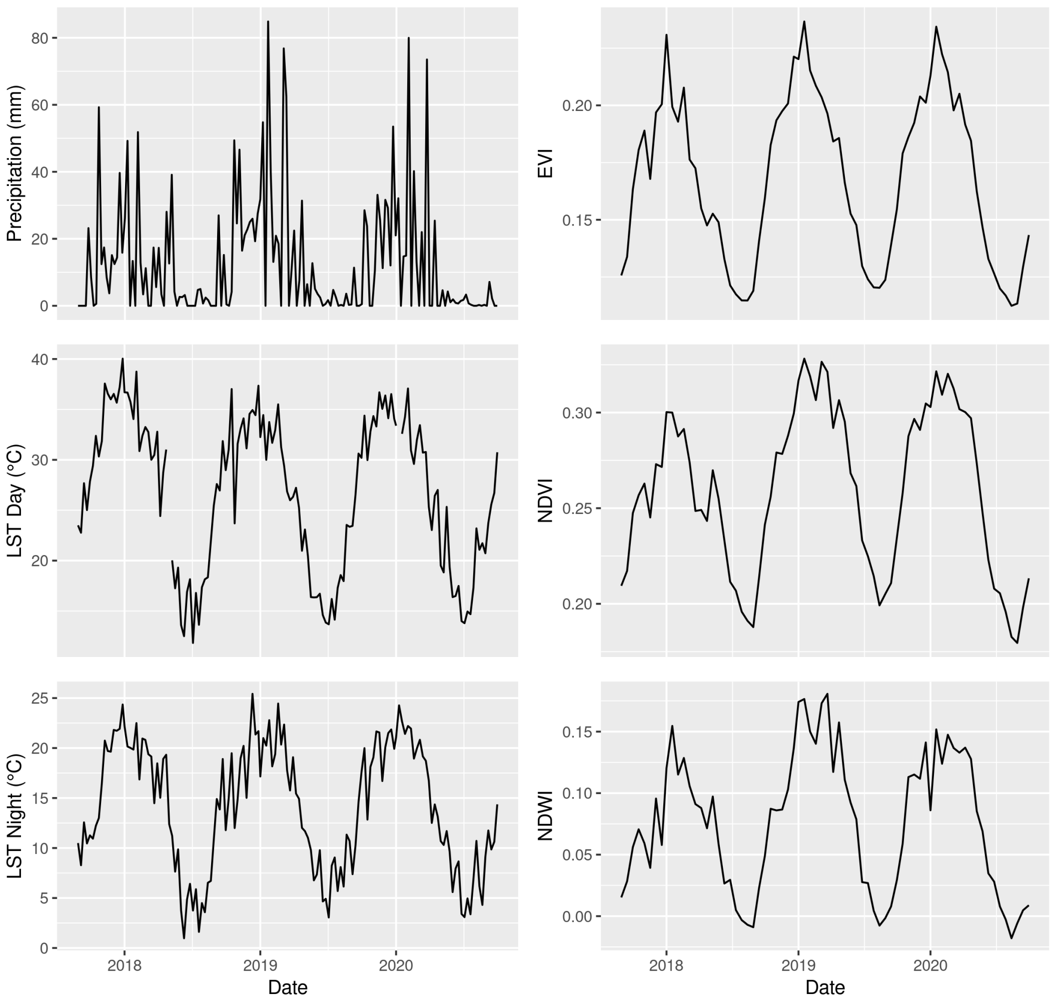

Figure S6. Time series of precipitation, LST day and night, EVI, NDVI and NDWI for the period 2017-2020 in Córdoba city, Argentina.

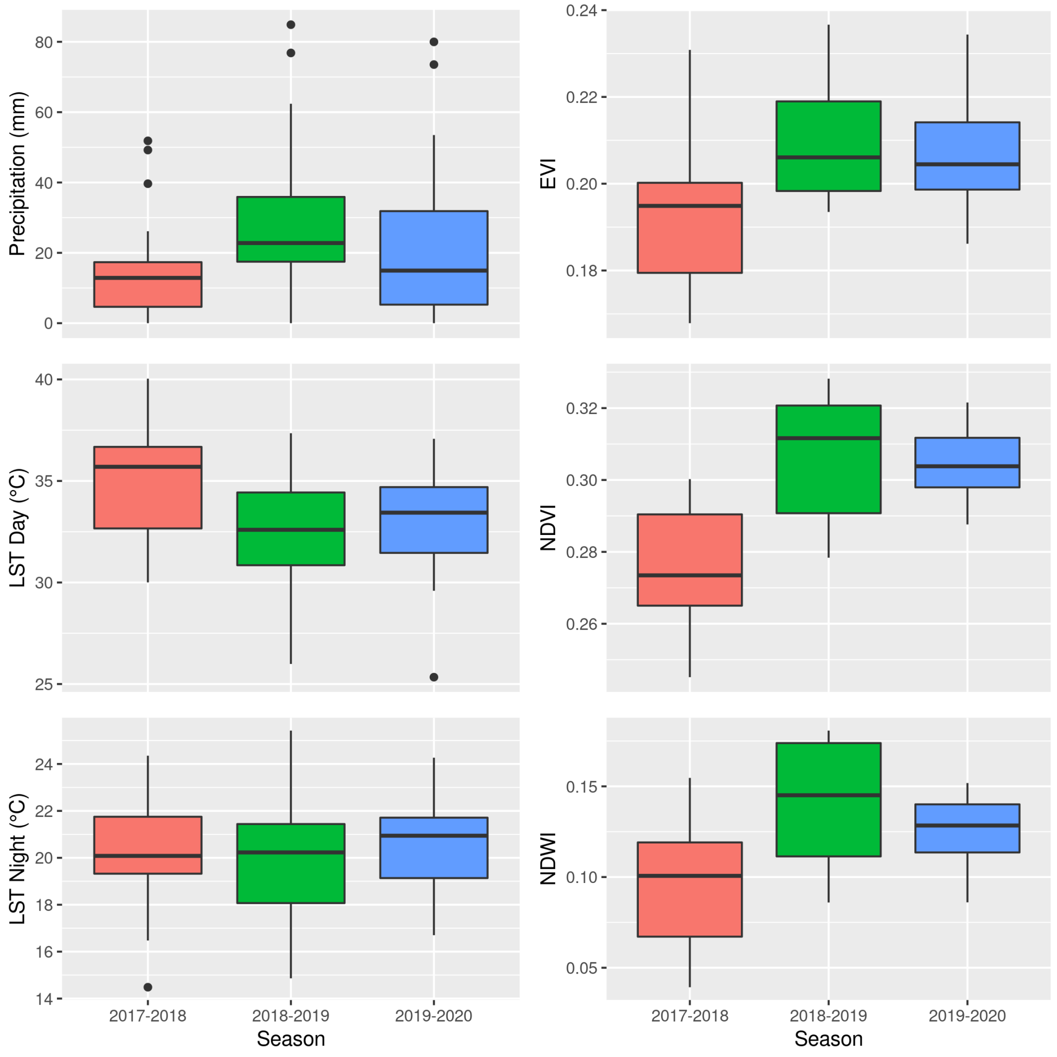

Figure S7. Comparison of precipitation, LST day and night, EVI, NDVI and NDWI for the core mosquito season (November to March) in the periods 2017-2018 and 2018-2019 and 2019-2020 in Córdoba city, Argentina.
